# Striving for group agency: threat to personal control increases the attractiveness of agentic groups

**DOI:** 10.3389/fpsyg.2015.00649

**Published:** 2015-05-27

**Authors:** Janine Stollberg, Immo Fritsche, Anna Bäcker

**Affiliations:** Department of Social Psychology, Institute of Psychology, University of LeipzigLeipzig, Germany

**Keywords:** social identity, control motivation, responses to threat, agency, group processes

## Abstract

When their sense of personal control is threatened people try to restore perceived control through the social self. We propose that it is the perceived agency of ingroups that provides the self with a sense of control. In three experiments, we for the first time tested the hypothesis that threat to personal control increases the attractiveness of being part or joining those groups that are perceived as coherent entities engaging in coordinated group goal pursuit (agentic groups) but not of those groups whose agency is perceived to be low. Consistent with this hypothesis we found in Study 1 (*N* = 93) that threat to personal control increased ingroup identification only with task groups, but not with less agentic types of ingroups that were made salient simultaneously. Furthermore, personal control threat increased a sense of collective control and support within the task group, mediated through task-group identification (indirect effects). Turning to groups people are not (yet) part of, Study 2 (*N* = 47) showed that personal control threat increased relative attractiveness ratings of small groups as possible future ingroups only when the relative agency of small groups was perceived to be high. Perceived group homogeneity or social power did not moderate the effect. Study 3 (*N* = 78) replicated the moderating role of perceived group agency for attractiveness ratings of entitative groups, whereas perceived group status did not moderate the effect. These findings extend previous research on group-based control, showing that perceived agency accounts for group-based responses to threatened control.

## Introduction

Group membership is important to people. It helps them to define who they are and to whom they belong, and it serves the satisfaction of basic human needs. The link between group membership and need satisfaction has been delineated for different motives, like self-esteem (Tajfel and Turner, 1979; Rubin and Hewstone, 1998), belongingness (Baumeister and Leary, 1995; Hornsey and Jetten, 2004), self-concept certainty (Jetten et al., 2004; Hogg, 2007), symbolic immortality (Greenberg et al., 1986; Castano et al., 2002), and, more recently, for personal control (Fritsche et al., 2013). Although, the need for control has been counted among the most basic human motives (Fiske, 2003; Pittman and Zeigler, 2007) only recently empirical research has begun to systematically test how group membership may satisfy the need for control (Fritsche et al., 2008, 2011, 2013). People need a general sense of control (White, 1959), they want to experience themselves as autonomous agents who are capable to exert influence on important aspects of their environment. We propose that a feeling of “having an impact on the world” is not limited to individuals (“I have an impact on”), but can be extended to social ingroups (“We have an impact on”), and that both contribute to the perception of the self as an agent. Accordingly, people can rely on their group membership to perceive themselves as agents when their sense of personal control is threatened (Fritsche et al., 2013). Building on research showing increased identification and group-based cognition following threat to personal control (Fritsche et al., 2008, 2013; Agroskin and Jonas, 2010, 2013), in the present article we aim at investigating what kind of groups work best for restoring a sense of control. Specifically, we tested the novel hypothesis that when a sense of personal control is threatened, people identify more strongly with agentic ingroups and find it more attractive to join agentic than less agentic groups.

People usually belong to many different groups that represent their social self (Tajfel and Turner, 1979). They identify with ingroups that are both accessible and salient in a particular context (Turner et al., 1987), and which may satisfy important self-related motives (Correll and Park, 2005). The need for control has been referred to as one of the most central human motives (White, 1959; Pittman and Zeigler, 2007). Deprivation of control can impair people's performance, well-being and health (Szpiler and Epstein, 1976; Rodin and Langer, 1977; Abramson et al., 1978), which is why people strive to uphold or restore a sense of control when control seems threatened (Rothbaum et al., 1982; Thompson et al., 1993). The model of group-based control (Fritsche et al., 2011, 2013) assumes that defining the self in terms of group membership (social identity; Tajfel and Turner, 1979) and joining in group-based action bolsters or restores people's sense of control through the (social) self when their sense of *personal* control is threatened. Initial empirical evidence has supported the group-based control approach: Salient lack of personal control increased ingroup bias (Fritsche et al., 2008, 2013), the expression of prejudice (Agroskin and Jonas, 2013; Fritsche et al., 2013; Greenaway et al., 2014a) and conformity to salient ingroup (but not to outgroup) norms (Stollberg et al., Unpublished manuscript). The reported control threat effects were obtained for different ingroups, ranging from classic social categories like nationality, and students, to work groups. However, it is an open question, whether people deprived of personal control support and identify with *any* ingroup. The model of group-based control suggests that people prefer groups that appear as (collective) agents and that could equip the self with a sense of (collective) control. This novel hypothesis is tested in the present research for both, identification with present ingroups and perceived attractiveness of joining new groups.

What are the properties of groups that give people a sense of collective control? According to Skinner (1996), personal control comprises the idea that the self as an agent can affect end states through instrumental actions (means). In a related vein, Preston and Wegner (2005) proposed that people are motivated to think of themselves as agents (“ideal agency”). According to them, agency is experienced for voluntary actions and can be traced back to three features: an intention to act, the free will to exert the action, and performing the action itself. The concept of agency closely resembles and helps to specify the foundations of control. Thus, drawing on both Skinner, and Preston and Wegner, we define a sense of control as *the perceived potential to affect important aspects of the environment through the autonomous self*. That is, perceptions of agency are essential for perceiving control, and striving for perceptions of the self as agentic should be a means to (re-) establish a perception of control.

Building on social identity principles (Tajfel and Turner, 1979), we propose that perceptions of agency are not limited to the personal level of the self, but can also describe the social level of the self. That is, in situations where the personal self is salient, people infer control from their sense of *personal* agency, whereas when a social self is salient, people's sense of control should be determined by perceptions of *collective* agency. In cases when personal agency and control seem thwarted, people may thus be motivated to define the self in terms of an agentic ingroup or to become a member of an agentic group. Applying the components of personal agency specified by Preston and Wegner (2005) to a collective agent (i.e., a social group) means that, for instance, a group of colleagues voluntarily cleaning up a messy meeting room would be perceived as agentic, because they are actually cleaning (perform action), they have planned to do so (collective intention), because they were annoyed by all the mess (free will). If people are motivated to reestablish a sense of control and to perceive themselves as autonomous collective agents, they should prefer groups that best represent all features of an ideal agent. First evidence that groups differ with regard to how well they satisfy agency needs was reported in research on lay people's group typologies (Lickel et al., 2000), showing that only task groups were associated with agency (Johnson et al., 2006). In a similar vein, Deaux et al. (1995) found that vocational identities were seen as more agentic than other identities.

Entitativity—the perception of a group as a real entity (Campbell, 1958; Yzerbyt et al., 2000; Brewer et al., 2004)—should be a necessary precondition to ascribe perceptions of agency to a group. Entitativity was linked to various group properties, such as boundedness, common fate, similarity of group behavior, homogeneity of group members, and sharedness of goals (Yzerbyt et al., 2000; Brewer et al., 2004). However, in most empirical studies, entitativity was understood only in terms of group homogeneity (Yzerbyt et al., 2000; Castano et al., 2003; Hogg et al., 2007) or within-group similarities (Lickel et al., 2000; Johnson et al., 2006; Crawford and Salaman, 2012). It was shown that identification with homogenous and bounded groups increased following self-uncertainty (Hogg et al., 2007), supporting the notion that groups with clearly defined prototypes satisfy a need for self-certainty. However, entitativity (i.e., the perception of a group as coherent entity through a certain level of homogeneity and boundedness among group members) should not be sufficient for satisfying the need for control, although it might be a necessary condition for group-based control to emerge. First, individuals require a certain level of similarity and boundedness to be perceived as a group at all. Second, basic agreement on collective goals and representations of the environment seems necessary to engage in collective behavior (which is not true for unspecific homogeneity that is not related to group goals). Nevertheless, whether or not the group is pursuing goals and has formed them autonomously is independent of perceived consensus and thus entitativity, but it is basically a question of collective agency. Although, some authors included agency in the concept of entitativity (Brewer et al., 2004), for the sake of clarity, here we treat both concepts separately, proposing that entitativity is necessary but not sufficient for group-based control to emerge. We hypothesize that threat to control increases identification with ingroups and increases the attractiveness of new groups, when these groups are highly entitative and at the same time highly agentic. Further, we control for other group features that may be related to collective agency, such as group size, group power, or status to flesh out the unique effect of collective agency for increasing group identification and group selection under threat.

To test our hypothesis that threat to control increases the attractiveness of agentic groups we conducted three studies. In Study 1, we investigated the effect of a control salience manipulation on identification with different types of ingroups, intimacy groups, task groups, social categories and loose associations. We hypothesized that control threat increases identification only for those types of groups that are characterized by both entitativity and agency (i.e., task groups; Lickel et al., 2000). Moreover, we investigated whether increased group identification following threat indirectly strengthened perceptions of intragroup support and collective control. According to the model of group-based control (Fritsche et al., 2011, 2013), when people respond to threatened control through identification with agentic ingroups they should perceive an increase in perceived control through the (social) self, that is, on the group level. Perceptions of heightened collective efficacy and perceived support within the ingroup should thus be indicative of control experienced on a group level. In line with this assumption Drury and Reicher (2009) found that identification with a social movement increased group members' perceptions of empowerment and collective efficacy, and therefore maintained the belief that the ingroup can attain its goal collectively.

In Studies 2 and 3, we studied the attractiveness of groups that were not (yet) a part of participants' identity. In particular, in Study 2 we were interested in the factors that determine the attractiveness of small vs. large groups. Small groups have been associated with better group performance due to less procedural losses on coordination (Kravitz and Martin, 1986), and motivation, like social loafing (Latané, 1981), which might affect the efficacy of group goal pursuit and increases the perception of small groups as agentic and efficacious, as it has been found in social dilemma studies (Kerr, 1989). However, there is contrary evidence as well: participants, who were asked to interact with others as a representative of a majority group reported a higher sense of personal control than those who thought to represent a minority group (Guinote et al., 2006). Finally, the relation between size and group agency perceptions was questioned in a multilevel-analysis by Watson et al. (2001), who investigated the impact of several individual and group factors on collective efficacy beliefs and who did not find a relation between group size and collective efficacy beliefs of group members. The ambiguity of these results suggests that whether small or large groups are perceived as more agentic may vary from situation to situation. We hypothesized that following threat to personal control participants should be more strongly attracted to groups of that size they perceive as relatively more agentic. Other perceived group characteristics that do not represent full-blown agency, such as social power or unspecific homogeneity, are not expected to moderate the effects of personal control threat on relative group attractiveness.

In Study 3, we measured the three components of perceived ideal group agency for entitative and non-entitative groups, hypothesizing that control threat affects group attractiveness only when the group is entitative *and* highly agentic. Following a control salience manipulation, we assessed the attractiveness of entitative and non-entitative realistic groups that were displayed on pictures and measured perceived agency and perceived group status as possible moderators.

## Study 1

We conducted Study 1 to examine the effect of threatened control on identification with ingroups of different types. As we argued above, not all groups should restore a sense of control to the same degree. Those high in both, entitativity and agency should be preferred when people have the opportunity to choose between ingroups of different types. A taxonomy of group types published by Lickel et al. (2001) reflects the intuitive typing of groups by lay people. They differentiate between task groups (e.g., work teams), intimacy groups (e.g., families), social categories (e.g., nations) and loose associations (e.g., people waiting together at the bus stop), showing that these four group types provide different benefits for people: participants primed with adjectives linked to a need for affiliation (e.g., connectedness, belonging) listed significantly more intimacy groups, whereas for achievement related adjectives (e.g., success, competence) more task groups were selected, and for self-esteem related adjectives (e.g., identity, distinctiveness) more social categories were chosen (Johnson et al., 2006; Crawford and Salaman, 2012). None of the studies investigating need fulfillment by these four different group types has explicitly investigated a need for control as a driver of ingroup identification. However, it seems obvious that task groups are those that are most intimately associated with the notion of agency and therefore collective control as, unlike the other groups, it is their most primary purpose to act. Therefore, we expect an increase in identification following threat to control only for task groups, because these groups are primarily perceived as agentic groups.

In line with the notion of group-based control, increased identification with a task ingroup following threat to control should further result in increased perceptions of collective efficacy and within-group support. Collective efficacy directly expresses a sense of collective control and agency whereas within-group support facilitates effective group coordination and goal pursuit and may thus indicate collective control in an indirect fashion. Recent findings initially support this assumption, by showing that group identification increased people's perceptions of control, which in turn enhanced personal well-being (Greenaway et al., 2015). Thus, we tested for indirect effects of control threat on collective efficacy and within-group support, mediated via identification with task groups.

### Method

#### Participants and design

One hundred and one university students participated in the study. We excluded three participants who had guessed the aim of the study, and five who had participated in a similar experiment previously. Thus, the final sample consisted of 93 participants, 66 were female and 26 were male, with a mean age of 21.41 years (*SD* = 2.22), one person did not indicate age or sex. The experiment had a 2 Control Salience (high/low) × 4 Group Identification (task group/intimacy group/social category/loose association) design with repeated measurement on the last factor.

#### Procedure

Participants were recruited at the campus of a German university. After they had agreed upon participation, they received a questionnaire, which introduced the study as a survey on personality traits. Then, participants were exposed to a control salience manipulation, similar to a manipulation that has been used previously in control threat research (Whitson and Galinsky, 2008). In the low control salience condition, participants read the following instruction (instructions for the high control salience condition in parentheses): *Please think about an important situation in your life, in which you had no (full) control over the things going on. Please, try to remember exactly and imagine the event vividly! How did you feel right now? Now, describe the situation and your thoughts about it in the following lines. Please, take as much time as necessary!*

The control manipulation was followed by a German version of the PANAS (Watson et al., 1988) and a questionnaire on sleep- and awakening patterns, which served as a delay task. We included a delay task, because different kinds of threat have been shown to produce effects only after a short delay (Wichman et al., 2008; Burke et al., 2010; Fritsche et al., 2012). Afterwards, participants received descriptions of the four group types (task group, intimacy group, social category, loose association) according to Lickel et al. (2000). For each group type they were asked to identify an example group to which they belonged. Then, identification with the group, collective efficacy, and within-group support were assessed. The order in which the group types were presented was counterbalanced, resulting in four different versions of the questionnaire.

##### Identification

Participants rated their identification with each group on five items on a 4-point-scale (1 = *do not agree* to 4 = *agree*). Four were adopted from Henry et al. (1999): “I think of this group as part of who I am.”, “I see myself as quite similar to other members of the group.”, “I enjoy interacting with the members of this group.”, “Members of this group like one another.”, another item “I am happy to be a member of this group.” was added, α_(*task group*)_ = 0.83, α_(*intimacy group*)_ = 0.53, α_(*social category*)_ = 0.71, and α_(*loose association*)_ = 0.86^1^.

##### Collective efficacy

Then, participants completed four items (1 = *do not agree* to 4 = *agree*), assessing collective efficacy beliefs for each group: “Together we are strong.”, “We can achieve things collectively, one cannot achieve individually.”, “Nobody should think you cannot count on us.”, “Together we even come through hard times.”, α_(*task group*)_ = 0.82, α_(*intimacy group*)_ = 0.61, α_(*social category*)_ = 0.79, and α_(*loose association*)_ = 0.87.

##### Within-group support

Perceived support among group members was assessed with three items (1 = *do not agree* to 4 = *agree*) for each group, adapted from Zimet et al. (1988): “There is always a member of the group around when I am in need.”, “I get the emotional support and help I need from my group.”, “I can count on my group when things go wrong.”α_(*task group*)_ = 0.80, α_(*intimacy group*)_ = 0.77, α_(*social category*)_ = 0.87, and α_(*loose association*)_ = 0.84. After completing the experiment, participants were debriefed and thanked for participation and received a chocolate bar.

#### Results

We expected low control salience to increase identification with the task group, but not with other group types. Therefore, we conducted a 2 Control Salience (low/high) × Order of Group Types × 4 Group Identification (task group/intimacy group/social category/loose association) analysis of variance, with repeated measurement on the last factor (for cell values see Table 1). The general level of group identification differed marginally for the order of group type presentation, *F*_(3, 78)_ = 2.62, *p* = 0.06, η^2^ = 0.09. However, as it did not interact with control salience, we report the descriptive values of the analysis regardless of presentation order. The results showed different levels of identification, depending on group type, *F*_(3, 78)_ = 334.29, *p* < 0.001, η^2^ = 0.93. Participants reported the highest level of identification with intimacy groups, followed by social categories and task groups, with the lowest levels of identification with loose associations. More importantly, a significant interaction of control salience and group type suggests that control salience affected participants' identification with ingroups differently, depending on group type, *F*_(3, 78)_ = 3.18, *p* = 0.03, η^2^ = 0.11. In line with predictions, people with low perceptions of control reported higher levels of identification with the task group than people with high perceptions of control, *F*_(1, 80)_ = 6.12, *p* = 0.02, η^2^ = 0.07. There were no simple effects of control salience on identification with intimacy groups, social categories or loose associations, all *p*'s > 0.12.

**Table 1:** **Mean and standard deviation scores for identification with different group types as a function of control salience (Study 1)**.

	**Identification**							
	**Task group**		**Intimacy group**		**Social category**		**Loose association**	
	***M***	***SD***	***M***	***SD***	***M***	***SD***	***M***	***SD***
Low control salient	2.75	0.61	3.74	0.29	2.65	0.53	1.81	0.76
High control salient	2.46	0.69	3.83	0.21	2.67	0.58	1.66	0.55

##### Mediational analyses

**Figure 1:**
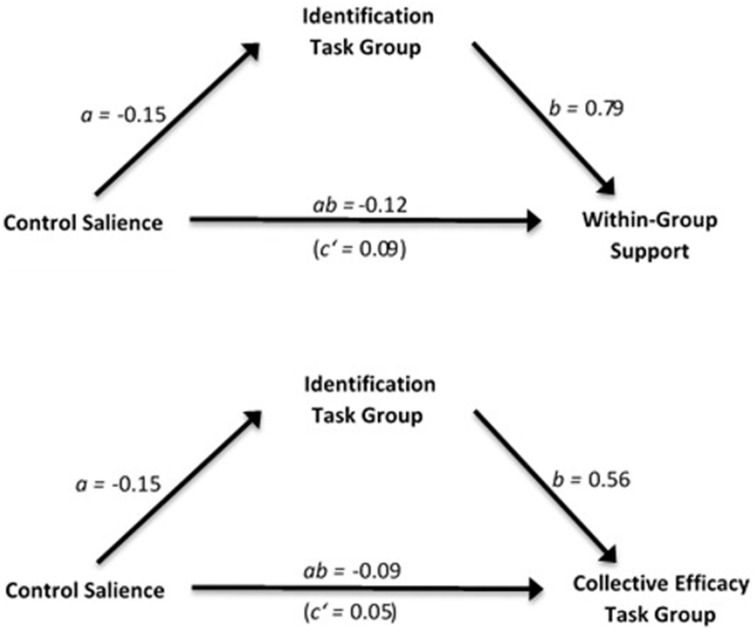
**Indirect effects of control threat in Study 1: Task group identification mediates the effect of control threat on both, perceived within-group support and collective efficacy perceptions**. Control salience was contrast coded: Low control (−1), high control (+1).

To test whether increased identification with a task group mediates the effect of control salience on collective efficacy beliefs and perceived within-group support from the task group, we conducted two separate simple mediation analyses (see Figure 1), using the macro *process* for SPSS (Hayes, 2013). Control salience was contrast coded for both analyses (low control = −1 vs. high control = 1). Participants in the low control salient condition showed more identification with their task group as participants in the high control salient condition (*a* = −0.15, *p* = 0.02), as identification increased in participants, collective efficacy beliefs increased too (*b* = 0.56, *p* < 0.001). A bias-corrected 95% confidence interval based on 2000 bootstrap samples for the indirect effect (*ab* = −0.09) was entirely below zero, CI [−0.18, −0.02], indicating an indirect effect. No evidence was found for a direct effect of control salience on collective efficacy beliefs (*c*′ = 0.05, *p* = 0.44).

Simple mediation analysis for within-group support revealed that low control salience increased participants identification with their task group as compared to high control salience (*a* = −0.15, *p* = 0.02), as identification increased, perceived group support increased too (*b* = 0.79, *p* < 0.001). Again, a bias-corrected 95% confidence interval (2000 bootstrap samples) for the indirect effect (*ab* = −0.12) was entirely below zero, CI [−0.23, −0.01]. No direct effect was found, (*c'* = 0.09, *p* = 0.15). Both reported mediations support our prediction that control threat indirectly increased collective efficacy beliefs, as well as perceived within-group support, via increased task group identification. As the control salience manipulation did not affect identification with another group type, we tested, whether there was a direct effect of control salience on collective efficacy beliefs and within-group support for the other group types. No direct effect could be observed for any of the two dependent measures, all *p*'s > 0.21.

#### Discussion

The results show that threat to personal control increased people's reports of identification with self-representative task groups, but not with other types of ingroups. This means that people under threat either identified more with task groups or more often choose task groups (but not other types of groups) they strongly identified with. Both possible processes express their motivation to uniquely associate the self with an ingroup, whose primary purpose is the active pursuit of a shared goal, as well as the perception to achieve the goal through joint effort, and therefore agency (Spencer-Rodgers et al., 2007). Identification with other groups that were also high on entitativity (Lickel et al., 2001), but rather low on agency, such as intimacy groups, was not affected by control threat. This supports our contention that only membership in entitative groups that at the same time is characterized by agency can restore feelings of perceived control through the collective self. Entitativity *per se* is not sufficient to increase the self-importance of a group following threat to control when more than one group is salient to people. While people identified highest with intimacy groups, which is in line with previous findings (Castano et al., 2003), self-importance of intimacy groups did not increase for people low in control, suggesting a specific mechanism of group-based control restoration. Nevertheless, previous studies also found increased support of other ingroups, such as nations, following threat to control (Fritsche et al., 2013). This could be explained by the fact that in all of these studies only one ingroup was salient to participants and thus only one option for experiencing collective control, whereas in the current study participants could choose that ingroup that appeared to be most appropriate for reestablishing a sense of control. Although in general, groups should heuristically be perceived as homogeneous agents (Brewer et al., 2004), the present study made salient the differences between the group types. Obviously, as the current results show, some ingroups are better suited to restore control perceptions than others, probably because they can provide a greater sense of agency.

The control restorative function of task ingroups is further supported by mediational evidence. The results show, as their sense of personal control decreased, people increased identification with a task group they belonged to, which in turn enhanced perceptions of collective efficacy for the task group. The same was true for perceived support among group members. Although, support by others may indicate less control through the self in interpersonal contexts, salient common group membership is likely to transform interpersonal support into an expression of the agentic “We,” and thus represents an indirect indicator of collective control. Hence, we conclude that people increase perceptions of collective control through identification with agentic ingroups, when control perceptions for the personal self are depleted. These findings illustrate that ingroup identification can in fact restore perceptions of control and may thus add to research examining beneficial effects of social identity for human well-being (social cure effects; Jetten et al., 2014).

However, agency is not the only characteristic of task groups and thus the effects of salient threat to personal control may be attributed to processes other than group-based control. To solve this issue, we conducted Studies 2 and 3, where we directly tested whether perceived group agency is necessary for increased group attractiveness following threatened personal control^2^. In Studies 2 and 3, we also intended to contrast the proposed moderating effect of perceived collective agency to the effects of other potential moderators, thereby turning from ingroup identification to the attractiveness of groups people do not (yet) belong. Here, we assume that potential new members become more likely to join the group when they perceive features of the group satisfying their personal needs (Moreland and Levine, 1982). Therefore, threat to personal control should increase the attractiveness of groups that are perceived as being agentic.

## Study 2

The results of Study 1 are a first indication that agentic ingroups are especially attractive for people who lack personal control. In Study 2 we extend this hypothesis to potential ingroups of which people are not part of yet. Furthermore, to test whether the effect of perceived collective agency is unique, possible effects of other factors, such as group size, perceived group power or perceived unspecific group homogeneity should be teased apart from agency perceptions.

In Study 2, we investigated the impact of control threat on attractiveness of groups differing in size. Small groups are better able to act in a coordinated manner, whereas in cases where large groups solve their coordination problems, as majority groups, they will have more social power to attain resources and could thus elicit effects on the environment. As thus, small and large groups' agency may be in the eye of the beholder, we decided to use relative measures, assessing participants' idiosyncratic perceptions of how agentic large groups are in comparison to small groups. In addition, we measured participants' relative perceptions of large vs. small groups on the possible group attribute moderators power and homogeneity, as well as group attractiveness as dependent variable. We expected participants to respond to threatened personal control by showing a relative preference for groups of that size they perceive as relatively more agentic. Although both group power and homogeneity could be related to perceptions of collective agency, they are each insufficient to cover all aspects of agency. Power can refer to personal influence, but also to the possession of resources and high status. Only the former perception shows a conceptual association with the outcome aspect of agency but less so with its process (shared intention and coordinated collective action). In a similar vein, homogeneity may prepare the ground for building shared intentions but is not implying that coordinated action or visible outcomes do occur. That is why we only expected full-blown agency to moderate control threat effects on group attractiveness.

### Method

#### Participants and design

Fifty university students participated in the study. We excluded one participant who had guessed the aim of the study, and two participants who participated in a similar experiment previously, resulting in a final sample of 47 participants. Twenty participants were female, and 27 were male, with a mean age of *M* = 27.81 years (*SD* = 7.95). A manipulation of control salience (high/low) served as independent variable. For both, dependent variable and moderators, we used measures that assessed participants' relative perceptions of large vs. small groups. We tested relative group agency, power, and group member homogeneity of large vs. small groups as a function of control threat on relative group attractiveness ratings.

### Procedure

Participants were asked at the campus of a German university to take part in a study on attitudes, biorhythm and personality. After they had agreed upon participation, they received a questionnaire, which opened with a control salience manipulation similar to those used in Study 1. In the low control salience condition (high control salience in parentheses) they read: *Take a moment to think about situations or incidents, in which you realized that you have very little (very much) control and impact on important things in your life. Please describe briefly in your own words one event or situation that made you feel helpless (influential). How did you feel in that situation?* After answering the two questions, participants were asked to indicate on a 7-point-scale (1 = *not at all* to 7 = *very much*), how much they felt in control over important aspects in their life in the situation they had just described, which served as a manipulation check. This was followed by a questionnaire on sleep- and awakening patterns, which served as a delay task, as in Study 1.

#### Group attractiveness

Then, participants made attractiveness rating for six pairs of groups, representing environmental organizations, political parties, companies, aid agencies, and cliques, that were each briefly described. Within one pair, the group descriptions differed only in group size (one small group, one large group). Participants were instructed to imagine that both groups would equally correspond to their attitudes and beliefs and that they should make a decision, which group they would rather like to join. To avoid a forced choice task, participants made ratings for each group of the pair with regard to the likelihood that they would join the group on a 7-point-scales (1 = *very unlikely* to 7 = *very likely*). To preserve the comparative nature of the judgments, we computed a difference score for the dependent variable of group attractiveness ratings, subtracting ratings of small groups from ratings of large groups, resulting in a variable that reflected the relative attractiveness of large compared to small groups.

#### Group attributes

Group attributes were assessed as relative measures, too. After group attractiveness ratings, participants were asked to indicate, which attributes large groups possess as compared to small groups: “Large groups are rather…” (1 = *powerless* to 7 = *powerful*); “Members of large groups are rather…” (1 = *dissimilar* to 7 = *similar*); “Large groups are rather…” (1 = *non-agentic* to 7 = *agentic*). After finishing the experiment, participants were thanked, fully debriefed and received a chocolate bar for their participation.

### Results

Participants in the low control salience condition indicated having perceived less control over important aspects in their life (*M* = 2.19, *SD* = 1.33) than participants in the high control salience condition (*M* = 5.40, *SD* = 1.24), *t*_(34)_ = −7.34, *p* < 0.001. Thus, the manipulation of control salience was successful.

**Figure 2:**
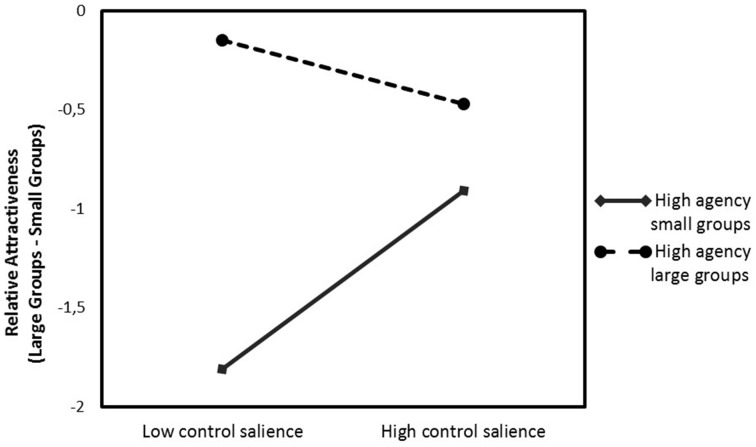
**Relative attractiveness of large groups compared to small groups (difference score) as a function of control salience (low vs. high) and relative agency perception of large vs. small groups**. Agency plotted at +1 SD (high agency large groups), and −1 SD (high agency small groups) about the mean (Study 2).

We tested whether control threat would increase relative preference for the group size that participants saw as relatively more agentic. For participants who saw large groups as more agentic than small groups, we predicted that the control threat would increase their relative preference for large groups. For participants who saw small groups as more agentic than large groups, we predicted the reverse, that control threat would increase their relative preference for small groups. Thus, we predicted a crossover interaction. We tested for moderation by perceived relative agency of large groups, using the *process* macro for SPSS by Hayes (2013). We regressed relative attractiveness of large vs. small groups on control salience (contrast coded with: high = 1, low = −1), perceived relative agency, and the Control Salience × Agency interaction. Agency and the interaction term were mean centered. The results showed that perceived agency moderated the effect of control salience on perceived relative attractiveness, indicated by a significant Control Salience × Agency interaction, *b* = −0.20, *t*_(44)_ = −1.81, *p* = 0.08 (see Figure 2). As simple slope analysis showed, when large groups were perceived as less agentic than small groups (−1 SD), the relative attractiveness of small groups increased under low control salience compared to high control salience, *b* = 0.45, *t*_(44)_ = 1.89, *p* = 0.07. No effect of control salience on relative attractiveness could be observed, when large groups were perceived as more agentic than small groups (+1 SD), *b* = −0.16, *t*_(44)_ = −0.68, *p* = 0.50. Looked at differently, participants in the low control salience condition perceived large groups as relatively more attractive than small groups, when large groups were perceived as more agentic (+1 SD), than when large compared to small groups were perceived as rather non-agentic (−1 SD), *b* = 0.55, *t*_(44)_ = 3.60, *p* < 0.001. In the high control salience condition, no effect occurred, *b* = 0.15, *t*_(44)_ = 0.92, *p* = 0.36.

We conducted another regression analysis, including all possible moderators (agency, power, and homogeneity), as well as the interaction terms of Control Salience × Agency, Control Salience × Homogeneity, and Control Salience × Power, to test for all moderators simultaneously. No moderation was found for power or homogeneity, *p*'s > 0.38. The initial interaction effect of Control Salience × Agency and the pattern of results remained the same.

### Discussion

The results of Study 2 confirm our hypothesis that agency, and not perceived power or group member homogeneity, moderates the effect of control salience on perceived attractiveness of potential ingroups. Threat to control increased the relative attractiveness of small groups when these were perceived as more agentic than large groups. For people with low perceptions of control, attractiveness of small compared to large groups was higher, when they perceived small groups to be relatively more agentic than large groups. However, control threat did not increase preference for large groups, when large groups were perceived as relatively more agentic than small groups. Perceived group power and member homogeneity did not moderate the control salience effect on group attractiveness. These findings emphasize the importance of agency as the crucial group feature that defines whether groups can serve as a resource for personal control.

Moreover, the results of the study provide first evidence that control threat can affect the choice of group membership. This could offer new insights, why some groups become attractive for people with low control perception and others do not. Although, perceived group power did not elevate control threat effects on group attractiveness, it is possible that groups high in power offer more opportunities to their members to perceive control than their low power counterparts. Power has been defined in terms of dependence, as an asymmetrical control over resources (e.g., Dépret and Fiske, 1999), but it has been also defined in terms of agency, as exerting control over the environment (van Dijke and Poppe, 2006). In intergroup research, collective action, the coordinated, voluntary action on behalf of the group, has been referred to as the basis for group power (Turner, 2005). This distinction between resource control and agency definitions of power may help to understand why perceived power is not related to control perceptions, as it seemed to be in the present study, and sometimes it is related to perceptions of control (Guinote et al., 2006). When groups are perceived as powerful due to *agency* reasons, they should have the potential to provide members with a sense of control. However, power due to *disposability of resources* does not necessarily imply that the group has acquired the resources through collective effort (high status groups often obtain resources because they are entitled but not because they have invested high effort) or that they apply these resources for common goal pursuit. Therefore, controlling resources does not necessarily express collective agency but may sometimes even indicate passivity (“being served”). This would support the finding from interpersonal power research that people often strive for personal power, which reflects the desire to increase one's sense of agency, but less so for social power, which reflects the desire to have control over others (van Dijke and Poppe, 2006).

## Study 3

In Study 2 perceived agency determined the effect of control threat on attractiveness ratings, however, as we used a global measure of agency, we were not able to assess whether all components of agency were present. It is possible that people indeed infer agency, but inference should be stronger when more components are present (Preston and Wegner, 2005). In Study 3 we thus used a more elaborated measure of collective agency perceptions, covering collective intention (cf. shared goals) and voluntary and active collective goal pursuit that represent important indicators of agency (Preston and Wegner, 2005). By employing realistic pictures of entitative and non-entitative groups we thought to use a more subtle procedure to present group features to participants. Groups were preselected as entitative or non-entitative, that means, the degree to which they were perceived to form a coherent entity (“groupness”). This allowed us to test directly our assumption that entitativity is necessary for perceptions of collective control, but that it is not sufficient to explain control threat effects on group attractiveness ratings. We expected personal control threat to increase attractiveness ratings of entitative *and* agentic groups, whereas attractiveness of groups that lack either entitativity or agency should not be affected by control threat.

Moreover, we tested whether the proposed effect of personal control threat on the evaluation of agentic groups is independent of the social status participants ascribe to the groups. This enabled us to test whether the effect is specific to processes of group-based control, which would imply a moderation by perceived agency but no parallel moderation by status. Alternatively, a moderation by status that cancels out the moderation by perceived agency would suggest a self-esteem account of the personal control salience effect, assuming that people strive for collective status in order to satisfy self-esteem needs (Tajfel and Turner, 1979; Rubin and Hewstone, 1998).

### Method

#### Participants and design

Eighty university students participated in the study, two participants were excluded from the sample, one guessed the aim of the study, and one participated in a similar experiment previously. Thus, the final sample consisted of 78 participants, 43 were female and 35 were male, with a mean age of 23.44 years (*SD* = 4.50). A manipulation of personal control salience (high/low) served as independent variable.

#### Procedure

Participants recruited at the university campus, were asked to take part in a study on personality traits and group perceptions. After participants had agreed upon participation, they received the questionnaire first containing a control salience manipulation, that has been previously used in group-based control research (Fritsche et al., 2013). Participants in the low control salience condition (high control salience in parentheses) read: *Take some moments to think of those aspects of your life, that give you a sense of helplessness and lacking impact (power and impact) on the important things of your life. Please, briefly jot down in your own words those three aspects of your life that make you feel most helpless (powerful)*^3^. As in Studies 1 and 2, this was followed by a delay task, the German version of the PANAS. Then, participants were presented with 12 pictures of an aggregate of people, six pictures depicted people forming an entitative group, whereas six other pictures depicted people in a similar context who did not form an entitative group (see Supplementary Material). The pictures were selected from a Pre-Study (*N* = 40), where we asked participants to estimate the degree to which the people displayed in the pictures constituted a real entity. The people in the entitative group pictures that we used in this study were perceived significantly more as a coherent entity than the people in the non-entitative pictures, *F*_(1, 39)_ = 179.30, *p* < 0.001. We told participants to imagine that they would like to start a similar group as it was depicted in the picture with people they like. We did so to ensure that participants perceived all groups as potential future ingroups. The pictures differed with regard to content, representing typical groups of the daily life. They displayed people in a seminar room, people in the streets of a city, people painting an artwork, people in an office room, people in a park, and people fishing. Then participants should rate each of the depicted groups on attractiveness of the group for the self, perceived agency, likeability of depicted group members, and perceived status.

##### Group attractiveness

Participants rated the attractiveness of the group for the self on four items: “I find the group attractive,” “I can nicely imagine myself being a part of a similar group,” “I think, the group members feel comfortable with their group,” “I would found a similar group myself.” Ratings were made on a 7-point-scale (1 = *not at all* to 7 = *absolutely*). Internal consistency of attractiveness ratings for each group ranged from α = 0.75 to α = 0.92. Group ratings were averaged over six pictures each, to build two composite score of general group attractiveness, one for entitative and one for non-entitative groups, which served as dependent variables.

##### Group agency

We created a five item measure of perceived agency to assess the three components of agency as we initially defined it: Sharedness of a common goal, voluntary group coordination to achieve the goal, and active pursuit of the goal. Items were: “The people in the group have a common goal that they are able to attain,” “I think, it is likely that the group will reach their common goal,” “The group is pursuing its goal collectively,” “The people in the group are actively working together,” “The group acts rather passively.”(*reverse coded*). Ratings were made on a 7-point-scale (1 = *not at all* to 7 = *absolutely*), internal consistency was good for all groups, ranging from α = 0.80 to α = 0.90, except for one group depicting people in a seminar room, with an internal consistency of α = 0.65. Again, ratings were averaged over six groups each, resulting in perceived agency of entitative groups and perceived agency of non-entitative groups.

##### Group status

Group status was measured with one item, on a 7-point-scale (1 = *not at all* to 7 = *absolutely*): “The group gives the impression of being held in high esteem.”, and averaged for entitative groups, α = 0.73, and non-entitative groups, α = 0.65.

##### Personal likeability

Further, we assessed perceived personal likeability of the individuals depicted in the pictures with one item (“The people in the group look sympathetic”). We intended to make sure that the depicted individuals did not a priori differ on personal likeability. As likeability ratings for people in entitative and non-entitative groups pictures did not differ for participants with low as compared to high perceptions of control, *p* = 0.32, we did not consider likeability in any further analysis. After finishing the experiment, participants were thanked, fully debriefed and received a chocolate bar for their participation.

#### Results

**Figure 3:**
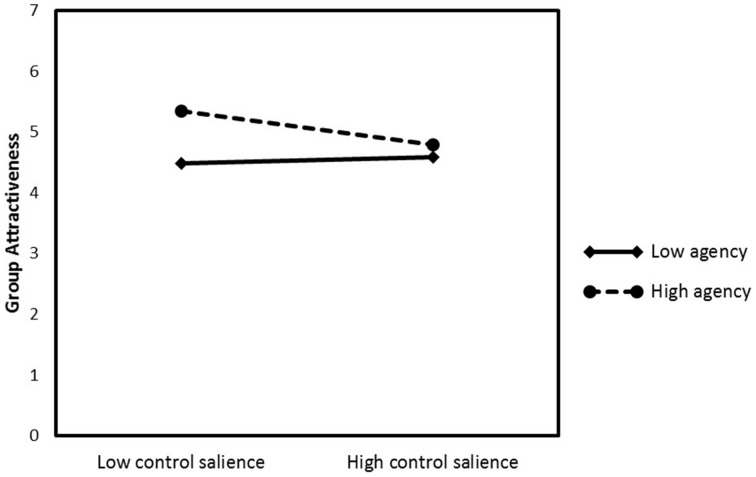
**Attractiveness ratings of entitative groups as a function of control salience (low vs. high) and agency perceptions (plotted at +/− 1 SD about the mean)**. Results are controlled for perceived group status (Study 3).

We expected low control salience to increase the attractiveness of entitative groups that at the same time are perceived as highly agentic. Thus, we conducted a moderation analysis, using the *process* macro for SPSS (Hayes, 2013). As control threat may affect attractiveness of entitative groups, depending on perceived group status, we tested for both moderations simultaneously (Model 2). Attractiveness of entitative groups was regressed on control salience (low = −1, high = +1), perceived agency of entitative groups, perceived status of entitative groups, Control Salience × Agency, and Control Salience × Status. Agency and Status, as well as both interaction terms were mean centered prior to the analysis. The results showed the predicted moderation for perceived agency, *b* = −0.23, *t*_(76)_ = −2.47, *p* = 0.02, and as expected no moderation for perceived status, *b* = 0.07, *t*_(76)_ = 1.08, *p* = 0.29. Simple slope analysis revealed that participants with low perceptions of control find entitative groups more attractive than participants with high perceptions of control, when they perceived the groups as very agentic (+1 SD), *b* = −0.28, *t*_(76)_ = −3.13, *p* = 0.003. When entitative groups were perceived as less agentic (−1 SD), attractiveness ratings were not affected by control perceptions, *b* = 0.05, *t*_(76)_ = 0.51, *p* = 0.62 (see Figure 3). Looked at differently, when low control was salient, very agentic groups were more attractive than less agentic groups, *b* = 0.59, *t*_(76)_ = 4.67, *p* < 0.001, whereas agency perceptions were not related to attractiveness ratings when high control was salient, *b* = 0.19, *t*_(76)_ = 1.45, *p* = 0.15. Thus, the results fully supported our hypothesis.

We conducted the same moderation analysis for non-entitative groups. Attractiveness of non-entitative groups was neither affected directly by control salience, nor did perceived agency or perceived status moderate the effect, all *p*'s > 0.41.

#### Discussion

The results of Study 3 further support our hypothesis that threat to control increases the attractiveness of groups that are perceived as both coherent entities and as highly agentic. Low control salience increased attractiveness ratings of entitative groups, when these groups were perceived as highly agentic. Of interest, perceived status did not moderate control threat effects on attractiveness ratings. Although, it should be acknowledged that, for economical reasons, we assessed group status with a single item measure, the results support our contention that perceived collective agency but not collective status accounts for the effects of personal control threat on group-based cognition and action. This enables us to distinguish processes of group-based control from processes of self-esteem maintenance, which should both represent central functions fulfilled by group membership.

### General discussion

Across three studies, we found converging evidence that threat to control increases the attractiveness of groups that are perceived as agentic, indicating that people try to restore and maintain their sense of control on the social level of the self when control is threatened for the personal self. Study 1 shows that threat to personal control increased identification only with task groups, but not with intimacy groups, social categories or loose associations. This supports our assumption that some groups are better suited to fulfill a need for control than others: when multiple ingroups are salient in a situation, people respond to control threat by increasing identification only with those groups that are both highly entitative and agentic (i.e., task groups). Entitativity in terms of “groupness” seems to be a necessary but not sufficient condition for group-based control as intimacy groups that are usually perceived as high in entitativity (Lickel et al., 2000) were not affected by control threat. Study 2 yielded more specific evidence that agency is the crucial group feature making groups attractive for group-based control restoration. Salient threat to personal control increased the relative attractiveness of small vs. large groups only when small groups were perceived as relatively more agentic than large groups. Other group features, such as perceived group power and perceived ingroup homogeneity did not moderate the effect of threatened control on group attractiveness ratings. This provides specific evidence for the moderating role of agency perceptions. Although, homogeneity and power may relate to specific components of agency perceptions they cannot be equated with agency. While similarity among group members may facilitate the generation of a shared group goal that increases agency perceptions, it does not imply joint goal pursuit and action. In a similar vein, group power usually allows for more opportunities and access to resources that could be used for effective goal pursuit, but powerful groups do not necessarily engage in goal achievement, because they might be satisfied with their current situation and behave rather passively. In Study 3, we conceptually replicated that perceived group agency moderates control threat effects on group attractiveness. Threat to control increased attractiveness ratings of entitative (but not of non-entitative) groups, when these were perceived as highly agentic, that is, when the groups were perceived as collectively and actively pursuing a common goal. Moreover, perceived group status did not moderate control threat effects on attractiveness ratings. The mere perception of high status or majority status (which is often used as a proxy for a high status group), is not sufficient to attract people with a deprived sense of control.

The present findings support the novel hypothesis derived from the model of group-based control (Fritsche et al., 2011, 2013), that people seek out for membership in and identification with agentic groups to restore a sense of control. Group membership satisfies different kinds of needs, but it is perceived collective agency that accounts for group-based control. Other group characteristics like homogeneity, power or group status did not affect group attractiveness ratings for control deprived people. Therefore, our findings extend previous research showing correlational evidence for differential need satisfaction by different groups (Johnson et al., 2006; Crawford and Salaman, 2012). They are also in line with research that considers the ingroup as a social resource (Correll and Park, 2005) for satisfying different needs, such as needs for certainty (Hogg, 2007), self-esteem (Rubin and Hewstone, 1998), and control (Fritsche et al., 2008). While, for instance, ingroup homogeneity can serve best a need for self-certainty (Hogg, 2007) or distinctiveness (Brewer, 1991; Jetten et al., 2004), perceived agency is crucial for control restoration. This adds to research showing that group features like agency or homogeneity are empirically related but functionally different constructs (Spencer-Rodgers et al., 2007; Crump et al., 2010). Whereas, entitativity understood in terms of groupness and homogeneity moderates the effect of a self-uncertainty threat on group identification (Hogg et al., 2007), our findings show that although entitativity seems necessary for group-based control, control deprived people only increase identification with those groups that are additionally perceived as agentic. Further research could investigate this more directly by manipulating group entitativity and group agency as independent between-subjects factors.

Although, the present findings support the notion that different threats elicit different reactions, they could also be understood in terms of a general threat and defense model (Jonas et al., 2014). This model assumes that threat causes a motivational discrepancy that could be resolved by approach-oriented reactions on the personal or social level. Increased attraction to ingroups following control threat represents such a distal defense mechanism on the social level. The present results showing identification with and attraction to agentic groups following threat support the notion that switching from behavioral inhibition to behavioral activation describes the threat defense process (Greenaway et al., 2014b). Future research should clarify the conditions under which people get to behavioral activation and regain a sense of agency and control either by engaging in personal or in social responses. The present research on group-based control indicates that threat influences social interactions on the group level as it determines people's sense of whom they belong and which groups they seek to join or to found.

#### People increase perceptions of collective control following personal threat through identification with agentic ingroups

Further evidence for the mechanism of group-based control comes from mediational analyses of Study 1. These findings are the first to show indirect effects of personal control threat on collective control through ingroup identification. This supports the assumption of group-based control that people can restore feelings of control through group membership. Although, previous research has demonstrated increased collective reactions to control threat (Fritsche et al., 2013), evidence that collective reactions in turn increase a sense of collective control has not been shown yet. Further, mediational evidence points to the specific benefits group membership has for people deprived of personal control: low personal control increased perceived social support by group members, mediated via ingroup identification. This complements research investigating the curative potential of social groups and the effects of ingroup identification on health and well-being (Haslam et al., 2009; Jetten et al., 2014). Previous findings showed the beneficial effects of identification on well-being following rejection and therefore threat to self-esteem (Branscombe et al., 1999) and the stress reducing effect of ingroup identification through perceived ingroup support (Haslam et al., 2009). More recently, Greenaway et al. (2015) found that perceived personal control mediated the beneficial effects of ingroup identification on personal well-being. The present findings support a control path on which the curative function of ingroup identification can unfold. They show that control threat enhances identification with agentic groups that in turn alleviate control loss: membership in agentic groups can help to restore and defend a threatened sense of control by providing group members with sense of collective control.

#### Limitations

In the present research, we compared a control threat salience condition with a condition in which high personal control was salient, without employing a neutral condition. Hence, it is possible that attractiveness of agentic groups does not increase following control threat, but decrease following control salience. However, we assumed the control threat condition to drive the effects, because in previous research control threat effects on ingroup defense emerged as compared to both, a high control condition and a neutral (i.e., dental pain) condition (Fritsche et al., 2008). No differences were observed between high control and the neutral topic. Nevertheless, future research would benefit from including a neutral condition to distinguish the control threat effects from possible effects of boosted control.

#### Implications for collective action and attractiveness of social movements

The finding that collective agency is the crucial feature that increases group attractiveness for people with low perceptions of personal control could help to explain why some groups gain members and support in society in times of threat and crisis while other groups do not (Fritsche et al., 2011). As perceived collective agency seems to restore a sense of control in people that are personally affected by societal crises, those groups that allow for perceptions of collective agency should gain most. Social movement organizations (Stürmer and Simon, 2004), such as gay-rights organizations or pro-environmental action groups are among those groups that are intimately associated with collective agency as they are set up for mastering collective tasks (i.e., they are true task groups). However, societal crises may also give rise to destructive forces and radicalized groups that become attractive for control deprived people when these groups unite against a common enemy thereby demonstrating collective agency through zealous protests or even violent actions. Summed up, there is reason to believe that, beyond perceptions of collective threat or disadvantage (van Zomeren and Iyer, 2009), collective action participation might be fertilized by threat to people's personal sense of control.

The possibility of increased collective action under conditions of threatened control shows that mechanisms of group-based control might be adaptive for resolving personal helplessness. This perspective adds to other recent research on social responses to control threat (Kay et al., 2008, 2009). Kay et al. (2008) have proposed that instead of trying to regain control through the (social) self, people may respond to threatened personal control by supporting external agents of control, such as God or the national government, and attributing control over bad outcomes to powerful enemies (Sullivan et al., 2010). This is thought to prevent a sense of randomness and lacking structure as external agents impose their order on the world. However, the present findings indicate that although personally helpless, people may first check out possibilities of extending the self to an agentic social ingroup that restores a sense of control through the self (primary control; Rothbaum et al., 1982) before they resort to external agents to preserve perceptions of order (secondary control; Rothbaum et al., 1982).

Our findings further imply which group people prefer when different groups are available. For control-deprived individuals groups focusing on similarity and similar appearance and less on coordinative effort in goal attainment, seem not to be first choice, if other groups exist that appear highly agentic. A group with a certain lifestyle, such as bohemianism, might be attractive to people because of the shared idea to live for art and love in an unorthodox, impoverished and unconventional manner, but that group will probably not be known for its agency, and might be therefore not primary for experiencing collective control. Instead, a successful political action group that actively fights for its goals may provide collective agency to potential members. This implies that the attractiveness of groups for new members could be emphasized by indications of agency. Groups that engage in collective doing, project planning or other forms indicative of concerted action should foster the perception of the group as agentic and allure people especially in times of personal threat.

In view of political extremism, deprivation of personal control may be added as another motivational determinant, in addition to self-uncertainty (Hogg, 2014), for engagement in political extremist groups that particularly consist of ideological or religious zealots that pursue group goals in a vigorous and tough-minded manner. Radicalized groups not only provide their members with a sense of a clear and distinct self that helps to reduce their self-uncertainty (Hogg, 2014) but in addition, radicalized groups can provide a sense of collective agency that helps to restore their sense of control. Violent extremist groups may stress their agency when they violate all norms of human co-existence as this highlights absolute commitment to a shared superordinate goal (it is even worth to violate all rules of conduct), free decision (against “social desirability” concerns), and the strength of active goal pursuit (which obviously cannot be prevented by others although they should be extremely motivated to stop the violations). These days, the rapid surge of extremist groups that attract foreigners from different countries who want to fight zealously for an “Islamic State” illustrates nicely the agentic potential these extremist and inhuman groups could offer. Personal feelings of lacking control may be one motivational factor that helps to explain why such vigorous campaigns gain support and new followers.

### Conflict of interest statement

The authors declare that the research was conducted in the absence of any commercial or financial relationships that could be construed as a potential conflict of interest.

## Footnotes

^1^Participants rated each group on warmth (warm, good-natured) and competence (competent, intelligent). Ratings were not affected by control salience manipulation, all *F* 's < 1. As we did not expect any difference on these dimensions, we did not consider it further.

^2^In the present study, however, we did not expect a moderation of the control threat effect by the measure of task group efficacy on identification. This is because we explicitly contrasted task groups with group types of lower agency that should have led to decreased variance on people's perceptions of task groups' agency (contrasted to the other groups, task groups are clearly agentic). Variance on perceived task group efficacy should have been primarily caused by the indirect effect of threat through task group identification instead, which appeared to represent a process of group-based control restoration.

^3^Please note, that the term “power” is used here as a synonym for “effectance” or “control” as it explicitly refers to objects of control that are not necessarily social. In German language the use of the word “power” [Macht] is not limited to social subjects, but can be used for non-social objects either. We used it in the present manipulation as in German everyday language the term “control” is not clearly associated with effectance.
