# Supplementary material for: Striving for group agency: threat to personal control increases the attractiveness of agentic groups
Source: Front Psychol. 2015 May 27;6:649. doi: 10.3389/fpsyg.2015.00649 (PMC4444748; doi:10.3389/fpsyg.2015.00649)
Supplement: Supplementary file 1 [file Table2.DOCX]

***Supplementary Material***

**Striving for Group Agency: Threat to Personal Control Increases the Attractiveness of Agentic Groups**

**Janine Stollberg_1_^1^*, Immo Fritsche_2_^1^, Anna Bäcker_3_^1^**

^1^Institute of Psychology, Department of Social Psychology, University of Leipzig, Leipzig, Germany

*** Correspondence:** Janine Stollberg, ^1^Institute of Psychology, Department of Social Psychology, University of Leipzig, Neumarkt 9-19, D-04109 Leipzig, Germany. janine.stollberg@uni-leipzig.de

**Keywords: social identity, control motivation, responses to threat, agency, group processes.**

1. **Table 2: Pictures of Entitative and Non-Entitative Groups**

*The table displays the pictures used in Study 3, including corresponding licenses and references.*

| **Topic** | **Entitative Group** | **Non-Entitative Group** |
| --- | --- | --- |
| People in a seminar room | 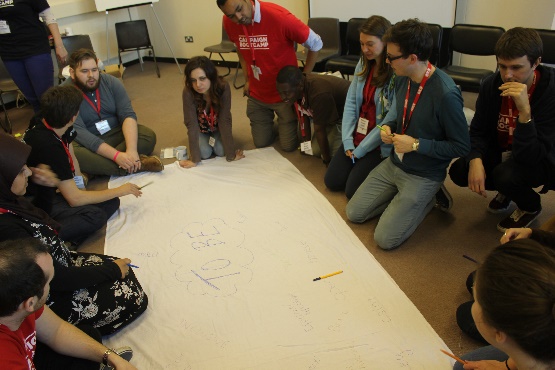  By Campaign Bootcamp (2014). Campaign Bootcamp2-Sunday. Used under Creative Commons License Attribution 2.0.  Retrieved from <https://www.flickr.com/photos/campaignbootcamp/13054802484/> | 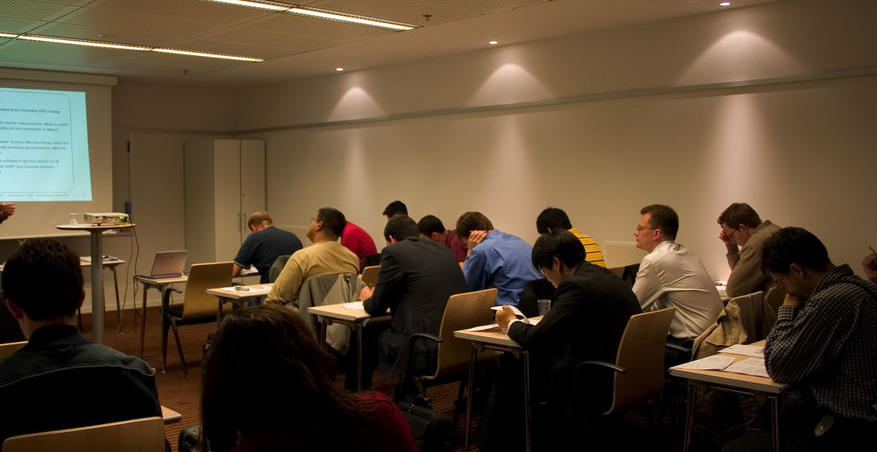  By McDiarmid, Alisdair (2005). Tutorials. Used under Creative Commons License Attribution 2.0. Retrieved from <https://secure.flickr.com/photos/alisdair/17301945/> |

| People in the streets of a city | 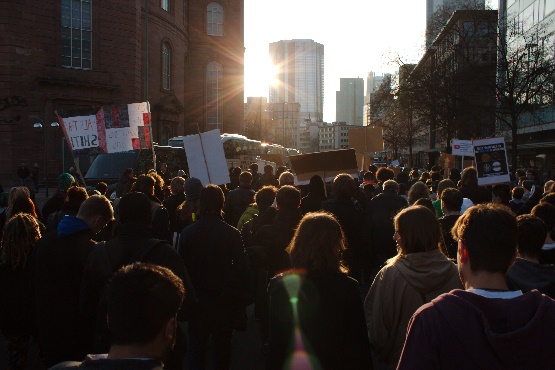  By Schmitt, Martin (2012). Demonstration gegen Acta. Used under Creative Commons License Attribution 2.0. Retrieved from <https://www.flickr.com/photos/foobarbaz/6928925699> | 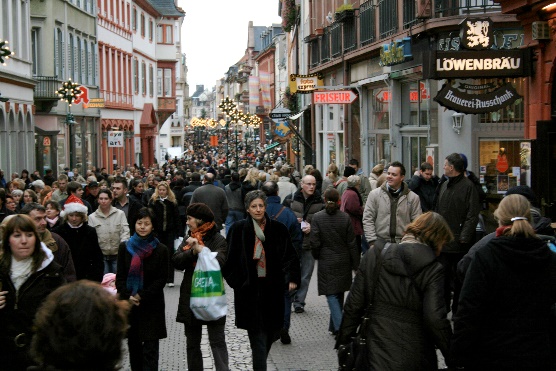  By Lademann, Frank (2007). Heidelberg im Advent. Used under Creative Commons License Attribution 2.0. Retrieved from <https://www.flickr.com/photos/coreforce/2097508788> |
| --- | --- | --- |
| People painting an artwork | 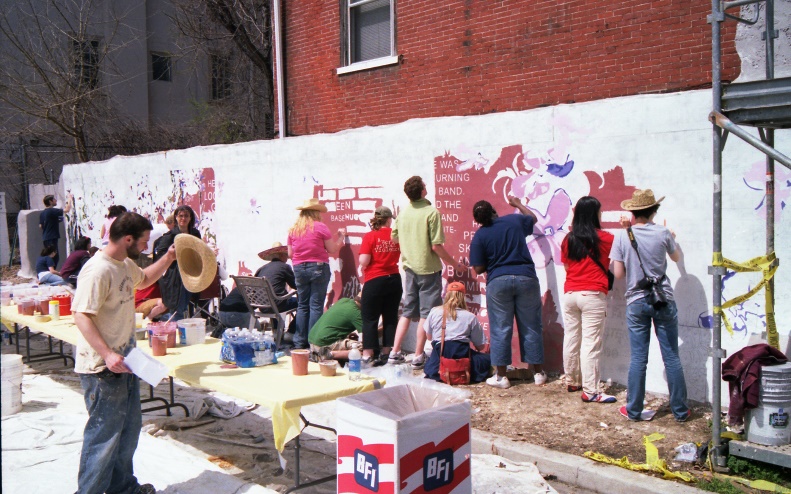  By Aquistbe (2008). Group Painting. Used under Creative Commons License Attribution-NonCommercial-NonDerivative 2.0. Retrieved from <https://www.flickr.com/photos/aquistbe/2432688918> | 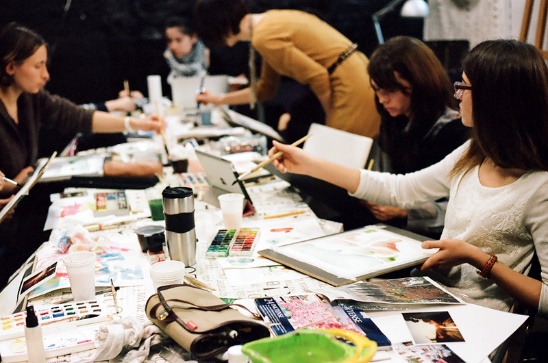  By Shoniya, Eka (2012). Used under Creative Commons License Attribution 2.0. Retrieved from <https://www.flickr.com/photos/eka_shoniya/8275672870/> |

| People in an office room | 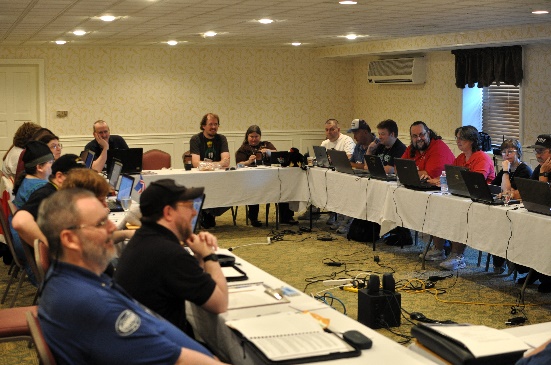  By Anbinder, Mark, H. (2011). EC/AB Meeting. Used under Creative Commons License Attribution-NonCommercial-ShareAlike 2.0. Retrieved from <https://www.flickr.com/photos/mhaithaca/6035678061> | 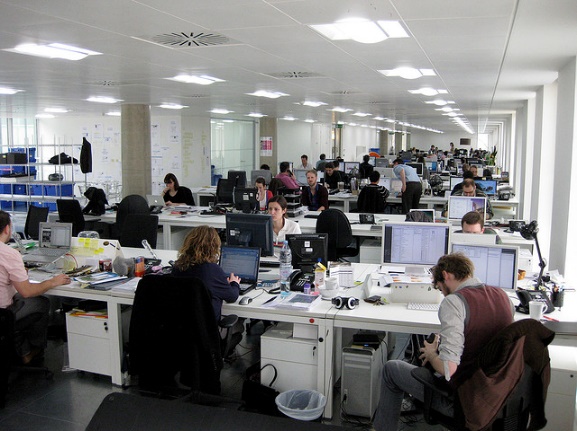  By Whitehouse, Phil (2009). New Office. Used under Creative Commons License Attribution 2.0. Retrieved from <https://www.flickr.com/photos/philliecasablanca/3344142642> |
| --- | --- | --- |
| People in a park | 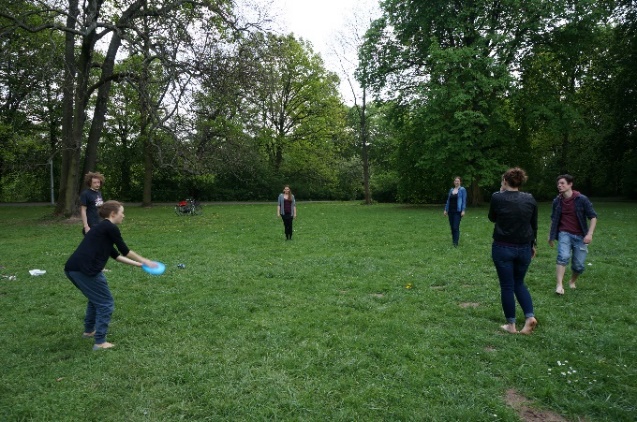  By Social Psychology Department, Leipzig. (2014). | 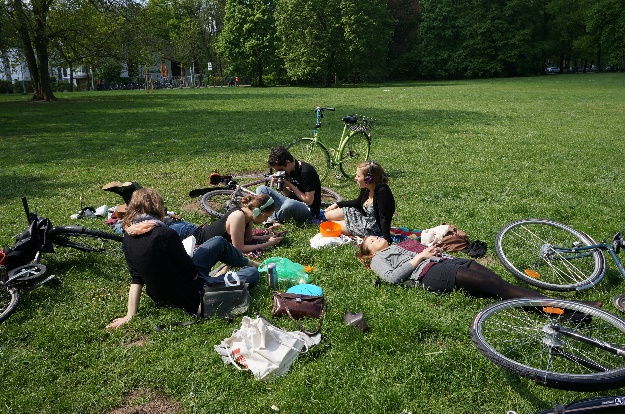  By Social Psychology Department, Leipzig (2014). |
| People fishing | 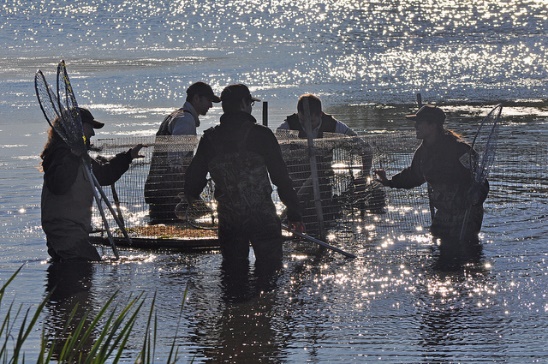  By U.S. Fish and Wildlife Service – Midwest Region. (2011). Teamwork. Used under Creative Commons License Attribution 2.0. Retrieved from <https://www.flickr.com/photos/usfwsmidwest/6214573130/> | 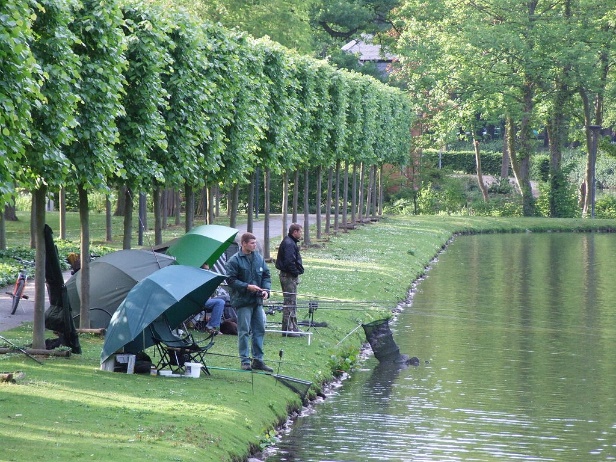  By Addicks, Johann, H. (2008). Angler am Kalkumer Schloss. Used under Creative Commons License Attribution 2.0. Retrieved from <https://commons.wikimedia.org/wiki/File:Angler_am_Kalkumer_Schloss_-_DSCF0217.JPG?uselang=de> |
